# Multiplex immunohistochemistry defines the tumor immune microenvironment and immunotherapeutic outcome in CLDN18.2-positive gastric cancer

**DOI:** 10.1186/s12916-022-02421-1

**Published:** 2022-07-11

**Authors:** Keren Jia, Yang Chen, Yu Sun, Yajie Hu, Lei Jiao, Jie Ma, Jiajia Yuan, Changsong Qi, Yanyan Li, Jifang Gong, Jing Gao, Xiaotian Zhang, Jian Li, Cheng Zhang, Lin Shen

**Affiliations:** 1 Department of Gastrointestinal Oncology, Key Laboratory of Carcinogenesis and Translational Research (Ministry of Education), Peking University Cancer Hospital & Institute, Beijing, China; 2 Department of Pathology, Key Laboratory of Carcinogenesis and Translational Research (Ministry of Education), Peking University Cancer Hospital & Institute, Beijing, China; 3 Panovue Biotechnology (Beijing) Co., Ltd, Beijing, China; 4 National Cancer Center/National Clinical Research Center for Cancer/Cancer Hospital & Shenzhen Hospital, Chinese Academy of Medical Sciences and Peking Union Medical College, Beijing, China

**Keywords:** Gastric cancer, CLDN18.2, Tumor microenvironment, Immune cell, Prognosis

## Abstract

**Background:**

The FAST study identified claudin-18 (CLDN18.2) as a promising novel therapeutic target for gastric cancer (GC). However, the tumor immune microenvironment and clinicopathological features of CLDN18.2-positive GC are unclear, making it difficult to develop and optimize CLDN18.2-targeted treatments.

**Methods:**

This study included 80 GC patients, 60 of whom received anti-PD-1/PD-L1 treatment. CD4/CD8/CD20/CD66b/CD68/CD163/PD-1/PD-L1/TIM-3/LAG-3/FoxP3/CTLA-4/HLA-DR/STING, and CLDN18.2 were labeled using multiplex immunohistochemistry (m-IHC) to decipher the rate and spatial distribution of T cells, B cells, macrophages, and neutrophils in formalin-fixed, paraffin-embedded tumor tissues isolated from these patients. Tumor immune-microenvironmental features and patient survival stratified by CLDN18.2 expression were analyzed using two independent-sample *t*-tests and log-rank tests, respectively.

**Results:**

We considered moderate-to-strong CLDN18.2 expression ≥ 40% of tumor cells as the cut-off for positivity. The proportion of CD8^+^PD-1^−^, CD8^+^LAG-3^−^, and CD8^+^TIM-3^−^ T cells was significantly higher in CLDN18.2-positive tumors than in negative tumors (0.039 vs. 0.026, *P* = 0.009; 0.050 vs.0.035, *P* = 0.024; 0.045 vs. 0.032, *P* = 0.038, respectively). In addition, the number of neutrophils (CD66b^+^) was higher in the CLDN18.2-positive group than in the negative group (0.081 vs. 0.055, *P* = 0.031, respectively), while the rates of M1 (CD68^+^CD163^−^HLA-DR^+^), M2 macrophages (CD68^+^CD163^+^HLA-DR^−^), and B cells (CD20^+^) were comparable between the CLDN18.2-positive and negative groups. The average numbers of CD8^+^PD-1^−^, CD8^+^LAG-3^−^, and CD8^+^TIM-3^−^T cells surrounding tumor cells within a 20-μm range were higher in CLDN18.2-positive tumors than in the CLDN18.2-negative tumors (0.16 vs. 0.09, *P* = 0.011; 0.20 vs. 0.12, *P* = 0.029; 0.18 vs. 0.12, *P* = 0.047, respectively). In addition, in the CLDN18.2-positive group, tumor cells surrounded by CD8^+^PD-1^−^, CD8^+^LAG-3^−^ T cells, or M1 macrophages within a 20-μm range accounted for a higher proportion of all tumor cells than those in the CLDN18.2-negative group (10.79% vs. 6.60%, *P* = 0.015; 12.68% vs. 8.70%, *P* = 0.049; 9.08% vs. 6.56%, *P* = 0.033, respectively). These findings suggest that CLDN18.2-positive GC harbors complex immune-microenvironmental features. Additionally, CLDN18.2-positive group had shorter OS and irOS than CLDN18.2-negative group (median OS: 23.33 vs.36.6 months, *P* < 0.001; median irOS: 10.03 vs. 20.13 months, *P* = 0.044, respectively).

**Conclusions:**

CLDN18.2-positive GC displayed unique immune-microenvironmental characteristics, which is of great significance for the development of CLDN18.2-targeted therapies. However, the impact of CLDN18.2-related microenvironmental features on prognosis requires further investigation.

**Supplementary Information:**

The online version contains supplementary material available at 10.1186/s12916-022-02421-1.

## Background

Gastric cancer (GC) is a leading cause of cancer-related deaths worldwide. GCs are usually diagnosed at a late stage, accompanied by distant metastasis and poor survival expectations. Although a series of molecular targets have been investigated in clinical trials, only HER2-targeted trastuzumab [1] and immune checkpoint inhibitors (e.g., pembrolizumab or nivolumab) demonstrated feasible response rates and have been documented as standard first-line therapies for advanced GC [2–4]. Consequently, to improve the survival of advanced GC, novel potential targets and development of additional precision medication regimens are urgently needed.

Claudins (CLDNs) belong to a family of tight junction proteins that mediate cell-cell adhesion and regulate selective permeability and ion homeostasis in epithelial cells [5]. CLDNs are also involved in the regulation of tumor proliferation and differentiation [5]. Encoded by the *CLDN18* gene, isoform 2 of claudin-18 (CLDN18.2) is buried in the tight junctions of gastric mucosal cells and is largely inaccessible to antibodies. However, as a result of malignant transformation and loss of cell polarity, CLDN18.2 becomes exposed on the surface of tumor cells [6]. These unique features draw attention to their role as potential therapeutic targets in GC.

Zolbetuximab is a novel chimeric monoclonal IgG1 antibody that binds to CLDN18.2, mediates tumor cell death, and induces immune effectors through antibody-dependent cellular cytotoxicity (ADCC) and complement-dependent cytotoxicity (CDC) [7, 8]. In the MONO study, zolbetuximab showed an objective response rate (ORR) of 9% as monotherapy in second- and subsequent-line therapy in GC [9]. In combination with chemotherapy, zolbetuximab induces pro-inflammatory cytokines. The FAST study enrolled patients with advanced gastric/gastro-esophageal junction and esophageal adenocarcinoma patients with moderate-to-strong CLDN18.2 expression. In the overall population, both progression-free survival (PFS) (hazard ratio [HR] = 0.44; 95% confidence interval [CI], 0.29-0.67; *P* < 0.0005) and overall survival (OS) (HR = 0.55; 95% CI, 0.39-0.77; *P* < 0.0005) were significantly improved with zolbetuximab + EOX compared with EOX alone [8]. The OS in FAST trial was comparable to that of HER-2 targeted therapy (FAST vs. ToGA: 13.0 months *vs*. 13.8 months) [10]. These encouraging results have led to SPOTLIGHT and GLOW phase III trials, which are currently recruiting participants. Considering the high specificity of CLDN18.2 that can facilitate tumor cells recognition by T cells [11], our group reported phase I results on Claudin 18.2-redirected chimeric antigen receptor (CAR)-T therapy (CT041) in gastrointestinal cancers (ClinicalTrials.gov, number NCT03874897) [12]. CT041 showed promising antitumor activity in patients with refractory CLDN18.2-positive gastrointestinal cancers, especially GC. In GC patients who had failed responding to at least two prior lines of therapies, CT041 achieved an ORR of 61.1% and median PFS of 5.6 months (95% CI, 2.6–9.2 months). These clinical trials emphasized the potential of CLDN18.2 to be the next therapeutic target following HER-2 in GC. However, further studies are warranted to identify the molecular traits of GC positive for CLDN18.2 expression, so as to boost the development of CLDN18.2-targeted therapies and strategies for combination therapy.

Recently, a number of retrospective studies have analyzed the clinicopathological characteristics of CLDN18.2-positive GCs [13–16]. In 134 Japanese GC patients, CLDN18.2 expression was significantly higher in Lauren diffuse classification and in high-grade (G3) tumors [17]. In a large Caucasian AGE/S cohort of 414 patients, high expression of CLDN18.2 was associated with neither histomorphological subtype, nor tumor state, nor OS [18]. However, these studies were conducted using traditional immunohistochemistry (IHC), and only provided superficial information. Probably due to the high heterogeneity in the GC microenvironment, the relationship between CLDN18.2 expression and its clinicopathological features is still debated.

As disclosed by current clinical trials, the combination of zolbetuximab with immunotherapy may stimulate T cell infiltration, subsequently enhancing the efficacy of immune checkpoint inhibitors [9]. Our trial CT041 also showed promising antitumor activity in patients with refractory CLDN18.2-positive GC [12], suggesting the involvement of CLDN18.2 with distinct immune features in the tumor microenvironment. In the tumor microenvironment, the subsets of immune cells and their precise locations in relation to cancer cells have demonstrated distinctive value in predicting tumor behavior [19]. Tumor-infiltrating immune cells are heterogeneous, exhibit functional and phenotypic plasticity, and may exert pro-tumorigenic and anti-tumorigenic effects [20]. Analyses of the relationships of individual tumor microenvironment components may advance the understanding of GC biology [21]. Recent developments in multiplex IHC (m-IHC) have enabled the simultaneous detection of multiple antigens in situ at single-cell resolution [22]. However, these methods have not yet been used to analyze the tumor immune microenvironment features related to CLDN18.2-positivity, and little is known about the impact of CLDN18.2 expression on immunotherapy in GC.

In this study, GC patients treated with immunotherapy were recruited to achieve two aims. As the primary aim, we investigated the association of CLDN18.2 expression with clinicopathological features and immunotherapeutic outcomes. As a secondary aim, we characterized the tumor microenvironment and spatial patterns of tumor-infiltrating immune cells in CLDN18.2-positive GC.

## Methods

### Patients

This study included 80 patients with GC who were treated at the Peking University Cancer Hospital between July 2014 and December 2019. The tissue samples of each patient before treatment were preserved using formalin-fixed, paraffin-embedded (FFPE) method. Pathologists (Y.S. and Y.H.) confirmed the histological classification of all tissue samples as adenocarcinoma. Patients with autoimmune diseases, HIV, or syphilis were excluded from the current study. This study was approved by the Ethics Committee of Peking University Cancer Hospital. All participants or their legal guardians signed informed consent forms. In accordance with the FAST clinical trial (NCT01630083), patients with ≥ 2+ membrane staining intensity in ≥ 40% of tumor cells were defined as CLDN18.2 positive, which helps to apply our findings to other clinical studies [8]. The mismatch repair (MMR) status was determined using IHC analysis of the expression of MLH1, MSH2, MSH6, and PMS2, which are DNA mismatch repair proteins. In situ hybridization (ISH) using a probe against Epstein-Barr-encoded RNA 1 (*EBER1*) was conducted to determine the EBV status. According to the RECIST criteria, patients with complete response (CR) or partial response (PR) were defined as responders, while those with progressive disease (PD) or stable disease (SD) were defined as non-responders. The interval between diagnosis and death or the last follow-up was defined as OS. The interval period between the start of immunotherapy and death or the last follow-up was defined as irOS. The interval between the start of immunotherapy and disease progression or the last follow-up was defined as irPFS.

### Multiplex immunohistochemistry

The expression intensity and spatial distribution of CLDN18.2, CD8, PD-1, TIM-3, LAG-3, CD4, FoxP3, CTLA-4, PD-L1, CD68, CD163, HLA-DR, STING, CD20, CD66b, and CD147 in tumor tissues and normal tissues surrounding the tumor were labeled using m-IHC. The tumor and adjacent tissues were fixed in formalin for 24–48 h within 30 min after being excised, and then dehydrated and embedded in paraffin using routine methods. The paraffin block containing the tumor or adjacent tissues was cut into sections with a thickness of 4 μm. FFPE tissue slides (4 μm) were melted and dehydrated at 60 °C for 12 h, and then deparaffinized and rehydrated using xylene and alcohol, respectively. The paraffin slides were placed in EDTA buffer (pH 9.0) or citrate buffer (pH 6.0) for FoxP3 staining, and the whole reactive system was placed in a microwave oven for heat-induced antigen retrieval. A commercially available blocking buffer (X0909; Dako, Santa Clara, CA, USA) was used to block the sections for 10 min. Additional file 1: Table S1 shows the antibodies used for the staining and their manufacturers. In accordance with the pre-optimized antibody concentration and the order of staining, the slides were incubated with the primary antibody and horseradish peroxidase-conjugated secondary antibody, and tyramine signal amplification (TSA) was performed. Antibody stripping and antigen retrieval were performed after each round of TSA. Then, 4′, 6-diamidino-2-phenylindole (DAPI, Sigma-Aldrich, St. Louis MO, USA; cat. D9542) was used to stain nuclei. Two experienced pathologists evaluated all stained GC specimens to ensure that they met the requirements for further analysis.

### Multispectral imaging and region of interest selection

The stained FFPE tissue sections were scanned using the Mantra quantitative pathology imaging system (PerkinElmer, Waltham, MA, USA) to obtain bright field and fluorescence images of the whole slides. Two pathologists observed the scanned pathological images using Phenochart (PerkinElmer) software and segmented the tumor core (TC) and normal (N) regions. The tumor core region and the normal region are not directly adjacent but are separated by the invasion margin (IM, the region between the two is approximately 1–1.5 mm wide). Subsequently, a fixed-size stamp (930 × 700 μm; × 20 objective lens) was used to select the representative region of interest (ROI) in the tumor center and normal regions. As many nonoverlapping ROIs as possible were chosen in each slice. Two pathologists were responsible for controlling the quality of the stained regions to ensure that all ROIs were within a suitable range of signal intensity.

### Recognition of cell morphology and spatial distribution

The feature extraction of multispectral images was performed using the inForm image analysis software 2.4 (PerkinElmer). The target protein was labeled with a special antibody and presented as a fluorophore on single-stained glass slides. A spectral library was established based on these fluorophores. The autofluorescence spectra of the tissues were extracted from the unstained sections. Based on the single-stained glass slides of each fluorophore, a spectral library was established to provide references for cell phenotypes. The characteristics of the cell phenotype included the characteristics of the fluorophore and the morphological characteristics of the nucleus (labeled by DAPI). Based on images containing a single fluorescence signal in the spectrum library, the inForm software extracted cell phenotype characteristics and identified each DAPI-stained cell in the mixed fluorescence view based on supervised methods. After the above procedures, the rate of each cell type in the tissue microenvironment (the percentage of the target cells in the total number of cells in the area) and the spatial distribution characteristics were counted and analyzed. Within a radius of 20 μm, the central cell and the surrounding cells were paired, based on which the networks including central tumor cells and surrounding immune cells were established. Two indicators quantifying these spatial networks were the effect score and effective percentage, which were defined as follows:

$$\mathrm{Effective}\ \mathrm{score}=\frac{\mathrm{number}\ \mathrm{of}\ \mathrm{edges}}{\mathrm{number}\ \mathrm{of}\ \mathrm{central}\ \mathrm{tumor}\ \mathrm{cells}}$$

$$\mathrm{Effective}\ \mathrm{percent}=\frac{\ \mathrm{number}\ \mathrm{of}\ \mathrm{central}\ \mathrm{tumor}\mathrm{s}\ \mathrm{cells}\ \mathrm{with}\ \mathrm{edges}}{\mathrm{number}\ \mathrm{of}\ \mathrm{central}\ \mathrm{tumor}\ \mathrm{cells}}$$

Effective score represents the average number of immune cells paired with tumor cells. Effective percent is the proportion of tumor cells that are paired with at least one immune cell in all tumor cells. Central and peripheral cells’ close proximity is essential for the binding of ligands and receptors mediating antitumor or immunosuppressive effects. Therefore, quantifying the relative spatial position by effective score and effective percent is helpful to understand the functions of cells in the tumor microenvironment.

### Gastric cancer cohort from The Cancer Genome Atlas database

The RNA sequencing data and matched clinical information from the GC cohort in The Cancer Genome Atlas (TCGA) were obtained from the UCSC Xena database [23]. The value of percent-splice-in (PSI) represents the percentage of a certain type of splicing event in all splicing events. For example, a PSI value of 0.8 indicates that the related splice isoforms account for approximately 80% of the transcripts in the sample. The PSI values corresponding to each GC sample were obtained from the TCGA SpliceSeq database, which could be used to select samples whose main splice isoforms of CLDN18 were CLDN18.2 [24]. The annotations of immune characteristics in the tumor microenvironment of the TCGA GC cohort were obtained from the TIMER 2.0 database [25, 26]. Only gastric adenocarcinoma samples that contained both RNA sequencing data and immune annotation information as well as PSI values greater than 0.9 were included in the subsequent analysis.

### Statistical analysis

The independent sample *t*-test and analysis of variance (ANOVA) were used to assess the association between tumor-infiltrating immune cells (TIICs) and clinicopathological characteristics. All correlation analyses were performed using Pearson’s method to calculate the correlation coefficient. The survival curves were constructed using the Kaplan-Meier method, and the comparisons of two survival curves were conducted using the log-rank test. Statistical analysis and visualization were performed using GraphPad Prism 9 or R version 4.1.0. All *P* values were two-tailed, and *P* < 0.05 was used to define statistical significance.

## Results

### Multiplex IHC assay to evaluate tumor microenvironment

**Fig. 1:**
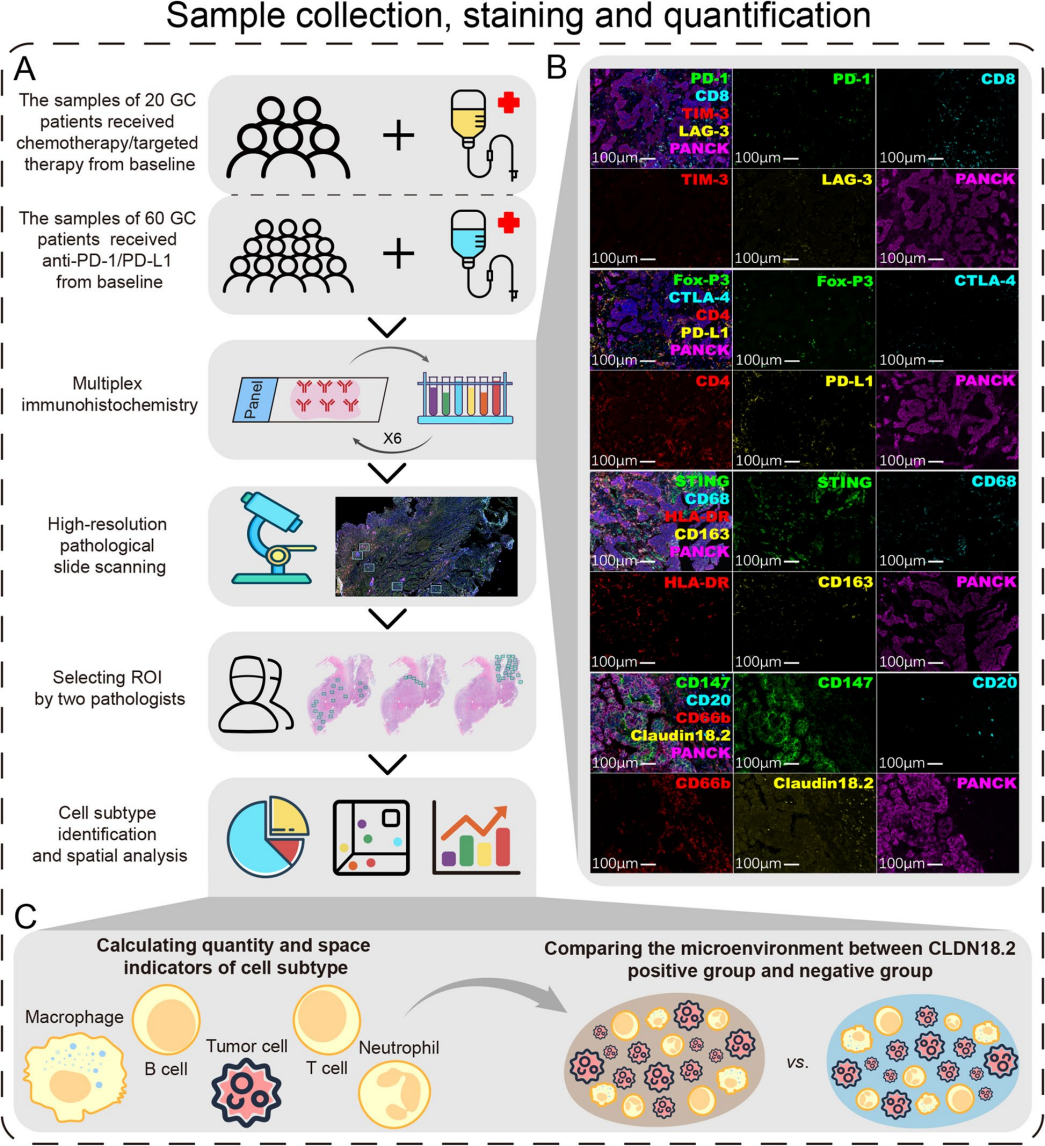
Rationale for the identification and characterization of GC’s immune microenvironment according to CLDN18.2 expression. **A** Study follow chart. **B** The merged and single-stained images for four representative panels of multiplex IHC. Scale bar: 100 μm. **C** Overview of the analysis design

To investigate the features of the tumor immune microenvironment in CLDN18.2-positive GC cancers, we quantified the rate and spatial distribution of immune cells in 80 full-face FFPE samples using m-IHC staining in whole tissue sections. Hematoxylin and eosin-stained tissue sections were also reviewed by two pathologists (Y.S. and Y.H.) to detect the TC, IM, and N areas, which we referred to as ROIs (Fig. 1A). Serially sectioned tissues were stained with m-IHC panels (Fig. 1B). A total of 6488 high-power fields (TC, 4477; IM, 993; N, 1,018) were imaged in all patients. A supervised image analysis system (inForm) was used to classify tumor and stromal segmentation and cell segmentation in CLDN18.2-positive and negative tumor tissues (Fig. 1C). We classified 73 immune cell subtypes according to the positivity and relative intensity of all markers in the individual panel.

### Correlation of CLDN18.2 expression with clinicopathological features and immunotherapeutic prognosis

The median age of all the patients was 60. All patients were Asian. Thirty-five tumors had poor differentiation, 22 moderate-poor differentiation, and 23 moderate differentiation. A total of 22 (27.5%) tumors were HER2-positive (Table 1). Mismatch repair deficiency was observed in 11 cases (13.7%), and a total of 10 (12.5%) tumors were positive for *EBER* ISH. According to PD-L1 expression, a combined positive score (CPS) ≥ 1 was found in 63 cases (78.7 %), while 46 patients (57.5%) presented a CPS ≥ 5. A total of 324 tumor samples with RNA sequencing data and immune annotation information were included in the study, and their PSI values were all greater than 0.9, which ensured that the main splice isoforms of CLDN18 in these samples were CLDN18.2. The clinicopathological characteristics of the TCGA GC cohort are summarized in Additional file 1: Table S2.

**Table 1:** The clinical, pathological, and molecular characteristics of CLDN18.2 positive or negative gastric cancer patients

Characteristic	All *N* = 80	Positive group *N* = 42	Negative group *N* = 38	*P* value
Age				0.53
Median, IQR	60 (54–66)	60.5 (54–66)	60 (50–66)	
Gender				0.19
Male	61 (76.3%)	35 (83.3%)	26 (68.4%)	
Female	19 (23.7%)	7 (16.7%)	12 (31.6%)	
ECOG PS				> 0.99
0	49 (61.3%)	26 (61.9%)	23 (60.5%)	
1	31 (38.7%)	16 (38.1%)	15 (39.5%)	
Location				0.66
GEJ	24 (30.0%)	14 (33.33%)	10 (26.3%)	
Non-GEJ	56 (70.0%)	28 (66.67%)	28 (73.7%)	
Lauren classification				0.18
Intestinal type	38 (47.5%)	16 (38.1%)	22 (57.9%)	
Diffused type	18 (22.5%)	12 (28.6%)	6 (15.8%)	
Mixed type	24 (30.0%)	14 (33.3%)	10 (26.3%)	
Stage				0.007
I	3 (3.8%)	1 (2.4%)	2 (5.3%)	
II	9 (11.3%)	0 (0%)	9 (23.7%)	
III	29 (36.2%)	17 (40.5%)	12 (31.6%)	
IV	39 (48.7%)	24 (57.1%)	15 (39.5%)	
Differentiation				0.010
Moderate	23 (28.8%)	6(14.3%)	17 (44.7%)	
Moderate-poor	22 (27.5%)	13(31.0%)	9 (23.7%)	
Poor	35 (43.7%)	23(54.8%)	12 (31.6%)	
HER2 expression				0.30
Positive	22 (27.5%)	9(21.4%)	13 (34.2%)	
Negative	58 (72.5%)	33(78.6%)	25 (65.8%)	
PD-L1 expression (CPS)				0.99
≥ 10	36 (45.0%)	19 (45.2%)	17 (44.7%)	
5–10	10 (12.5%)	5 (11.9%)	5 (13.2%)	
1–5	17 (21.3%)	9 (21.4%)	8 (21.1%)	
< 1	17 (21.3%)	9 (21.4%)	8 (21.1%)	
MMR status				> 0.99
pMMR	69 (86.3%)	36 (85.7%)	33 (86.8%)	
dMMR	11 (13.7%)	6 (14.3%)	5 (13.2%)	
EBV status				0.13
Positive	10 (12.5%)	8 (19.1%)	2 (5.3%)	
Negative	70 (87.5%)	34 (80.9%)	36 (94.7%)	
Treatment				0.12
Chemotherapy±targeted therapy	20 (25.0%)	14 (33.3%)	6 (15.8%)	
Anti-PD1/PD-L1 based therapy	60 (75.0%)	28 (66.67%)	32 (84.2%)	

*Abbreviations*: *IQR* interquartile range, *GEJ* gastroesophageal junction, *dMMR* deficient mismatch repair, *pMMR* proficient mismatch repair

**Fig. 2:**
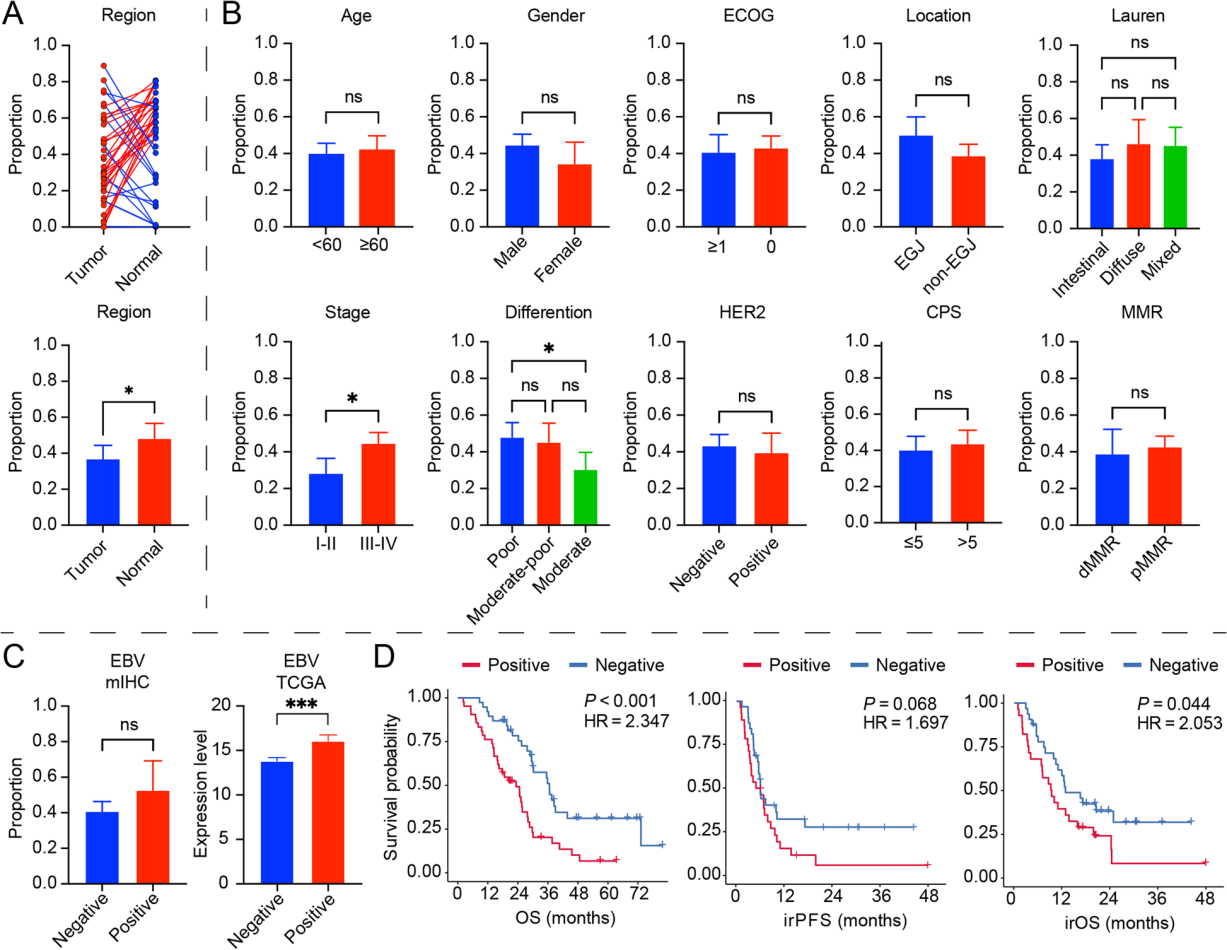
The association of CLDN18.2 positivity with clinicopathological and prognostic features in GC. **A** Comparison of CLDN18.2 expression between tumor and tumor adjacent normal area. Proportion: the proportion of CLDN18.2-positive tumor cells in the tumor region. Student *t*-test. * *P* < 0.05. ns: not significant. **B** The CLDN18.2 expression grouped by clinicopathologic features. Student *t*-test. * *P* < 0.05. ns: not significant. **C** The comparison of CLDN18.2 expression between EBV status in our cohort and TCGA cohort. Student *t*-test. *** *P* < 0.001. ns: not significant. **D** Overall survival, immunotherapy related OS, immunotherapy related PFS of patients based on CLDN18.2 expression in tumor core. Log-rank (Mantel-Cox) test. HR: hazard ratio

Representative stained tumor tissues are presented in Additional file 2: Fig. S1A-D according to the 0-3+ staining intensity classification. CLDN18.2 moderate-to-strong expression was also observed in normal gastric mucosa. We found that CLDN18.2 positivity showed a relatively higher trend in normal tissues than in tumor tissues (Fig. 2A). In accordance with the FAST clinical trial (NCT01630083), we defined ≥ 2+ membrane staining intensity in ≥ 40% of tumor cells as CLDN18.2-positive. In our cohort, CLDN18.2-positive expression was detected in 42 GC tissues [8]. CLDN18.2 expression correlated with higher stage disease (III, IV) at diagnosis (*P* = 0.03) and poor tumor differentiation (*P* = 0.02). Tumors located in the esophagogastric junction (GEJ) seemed to have a higher CLDN18.2 expression than non-GEJ tumors (*P* = 0.05). There was no association between CLDN18.2 expression and age, gender, ECOG status, HER2 status, PD-L1 CPS score, or MMR status (Table 2, Fig. 2B). In tumor-adjacent normal tissue, we also observed the same trend of higher CLDN18.2 expression associated with tumor differentiation and location (Table 2). EBV-positive GCs tended to show strong CLDN18.2 expression in all patients, which was consistent with the results in TCGA (Fig. 2C, Additional file 1: Table S2). Furthermore, we analyzed the prognostic value of CLDN18.2. The expression of CLDN18.2 (≥ 40%) was associated with an inferior OS (*P* < 0.001) in our cohort (Fig. 2D).

**Table 2:** The proportion of CLDN18.2 moderate-to-strong expression in clinical, pathological, and molecular subtypes

Characteristic	TC region	*P* value	Normal region	*P* value
Age				
< 60	0.408	0.64	0.499	0.75
≥ 60	0.434		0.471	
Gender				
Male	0.446	0.10	0.468	0.48
Female	0.344		0.524	
ECOG PS				
0	0.431	0.68	0.481	0.98
1	0.408		0.483	
Location				
GEJ	0.501	0.051	0.642	0.015
Non-GEJ	0.388		0.442	
Lauren classification				
Intestinal type	0.382	0.36	0.468	0.62
Diffused type	0.463		0.558	
Mixed type	0.454		0.448	
Stage				
I–II	0.283	0.028	0.371	0.20
III–IV	0.446		0.510	
Differentiation				
Moderate	0.303	0.015	0.488	0.042
Moderate-poor	0.453		0.337	
Poor	0.480		0.586	
HER2 expression				
Positive	0.395	0.54	0.510	0.67
Negative	0.432		0.471	
PD-L1 expression (CPS)				
≤ 5	0.402	0.53	0.465	0.71
>5	0.436		0.497	
MMR status				
pMMR	0.427	0.62	0.498	0.40
dMMR	0.388		0.335	
EBV status				
Positive	0.526	0.14	0.445	0.74
Negative	0.407		0.489	

*Abbreviations*: *IQR* interquartile range, *GEJ* gastroesophageal junction, *dMMR* deficient mismatch repair, *pMMR* proficient mismatch repair

In GC patients who received anti-PD-1/PD-L1 immunotherapy, CLDN18.2 showed a poor area under the curve value for predicting treatment response (Additional file 2: Fig. S2A). However, we observed a negative prognostic effect of irPFS (*P* = 0.068) and irOS (*P* = 0.044) (Fig. 2D). The clinicopathological characteristics of the 60 GC patients who received anti-PD-1/PD-L1 immunotherapy are summarized in Additional file 1: Table S3. In addition, because CLDN18.2 showed high expression in normal tissue, we analyzed the prognosis according to normal tissue and the comparison of CLDN18.2 between tumor and matched normal tissue. We did not find survival differences in the normal tissue or the comparison groups (Additional file 2: Fig. S2B-C).

### Immune-microenvironmental features in tumor/normal tissues of CLDN18.2-positive GC

To evaluate the tumor-immune microenvironment in CLDN18.2-positive GC, we performed a comparison of immune cell subtypes based on CLDN18.2 expression.

**Fig. 3:**
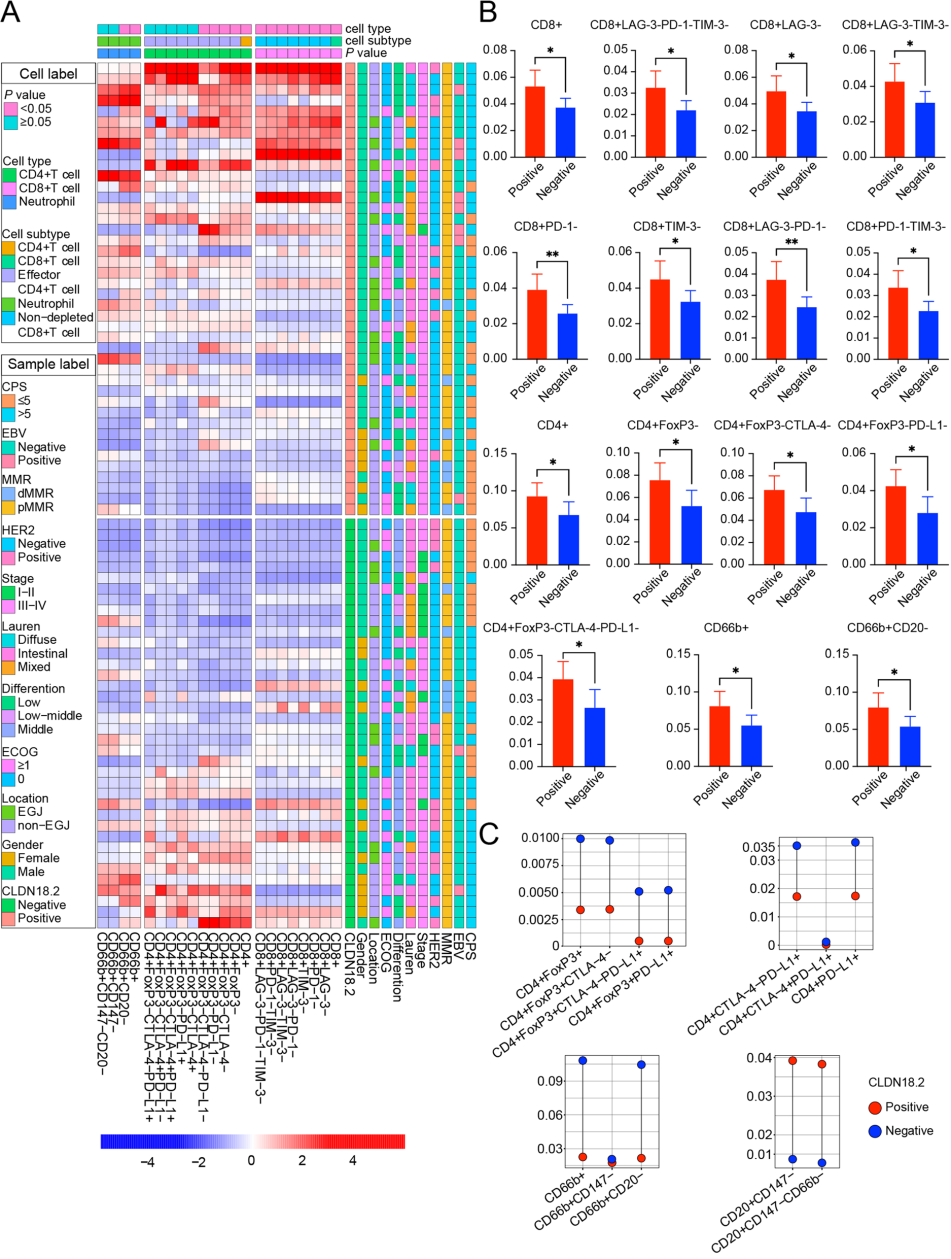
Unique immune cell subtypes in CLDN18.2-positive GC and normal tissues. **A**, **B** The rate of immune cell subtypes in tumor core based on CLDN18.2 expression and other clinicopathological features. Blue bars: CLDN18.2-negative; Red bars: CLDN18.2-positive. *P < 0.05, **P < 0.01, ***P < 0.001 and not significant (ns). Student *t*-test. **C** The rate of immune cell subtypes in tumor-adjacent normal regions grouped by CLDN18.2 expression. Blue dots: CLDN18.2-negative; Red dots: CLDN18.2-positive. * *P* < 0.05, ** *P* < 0.01, *** *P* < 0.001 and not significant (ns). Student *t*-test

We analyzed the correlation between CLDN18.2 and other immune checkpoint inhibitors in the tumor core; unfortunately, we did not observe any correlation with PD-1, PD-L1, CTLA-4, LAG-3, or TIM-3 (Additional file 2: Fig. S3). In the tumor core, we found that the CLDN18.2-positive group showed significantly higher total CD8^+^ T cells, CD8^+^LAG-3^−^ T cells, CD8^+^PD-1^−^ T cells, and CD8^+^TIM-3^−^ T cells than the CLDN18.2-negative group did (0.053 vs. 0.037, *P* = 0.023; 0.039 vs. 0.026, *P* = 0.009; 0.050 vs.0.035, *P* = 0.024; 0.045 vs. 0.032, *P* = 0.038, respectively). As shown in Fig. 3A-B, total CD4^+^ T cells, CD4^+^FoxP3^−^ T cells, CD4^+^FoxP3^−^CTLA-4^−^ T cells, and CD4^+^FoxP3^−^PD-L1^−^ T cells were significantly higher in the CLDN18.2-positive group than in the CLDN18.2-negative group (0.093 vs. 0.068, *P* = 0.045; 0.076 vs. 0.052, *P* = 0.026; 0.068 vs. 0.048, *P* = 0.024; 0.043 vs. 0.028, *P* = 0.020, respectively), detailed information of which were stored in Additional file 1: Table S4. Neutrophil levels were also significantly higher in the CLDN18.2-positve group (0.081 vs. 0.055, *P* = 0.031). Association of higher CD8^+^ T cells and neutrophils with CLDN18.2-positive tumors in our cohort was consistent with that in TCGA (Additional file 2: Fig. S4A). However, we did not observe a significant difference in macrophages between the CLDN18.2-positive and negative groups (Additional file 2: Fig. S4B-D). The lack of PD-1/PD-L1-positive lymphocytes in CLDN18.2-positive GC limited its chance to benefit from PD-1/PD-L1 inhibitors, while infiltrating neutrophils may also augment this negative therapeutic response. Thus, our findings suggest that CLDN18.2-positive GC is unlikely to benefit from PD-1/PD-L1 inhibitors.

### Spatial organization of tumor and immune cells associated with CLDN18.2-positivity in gastric cancer

**Fig. 4:**
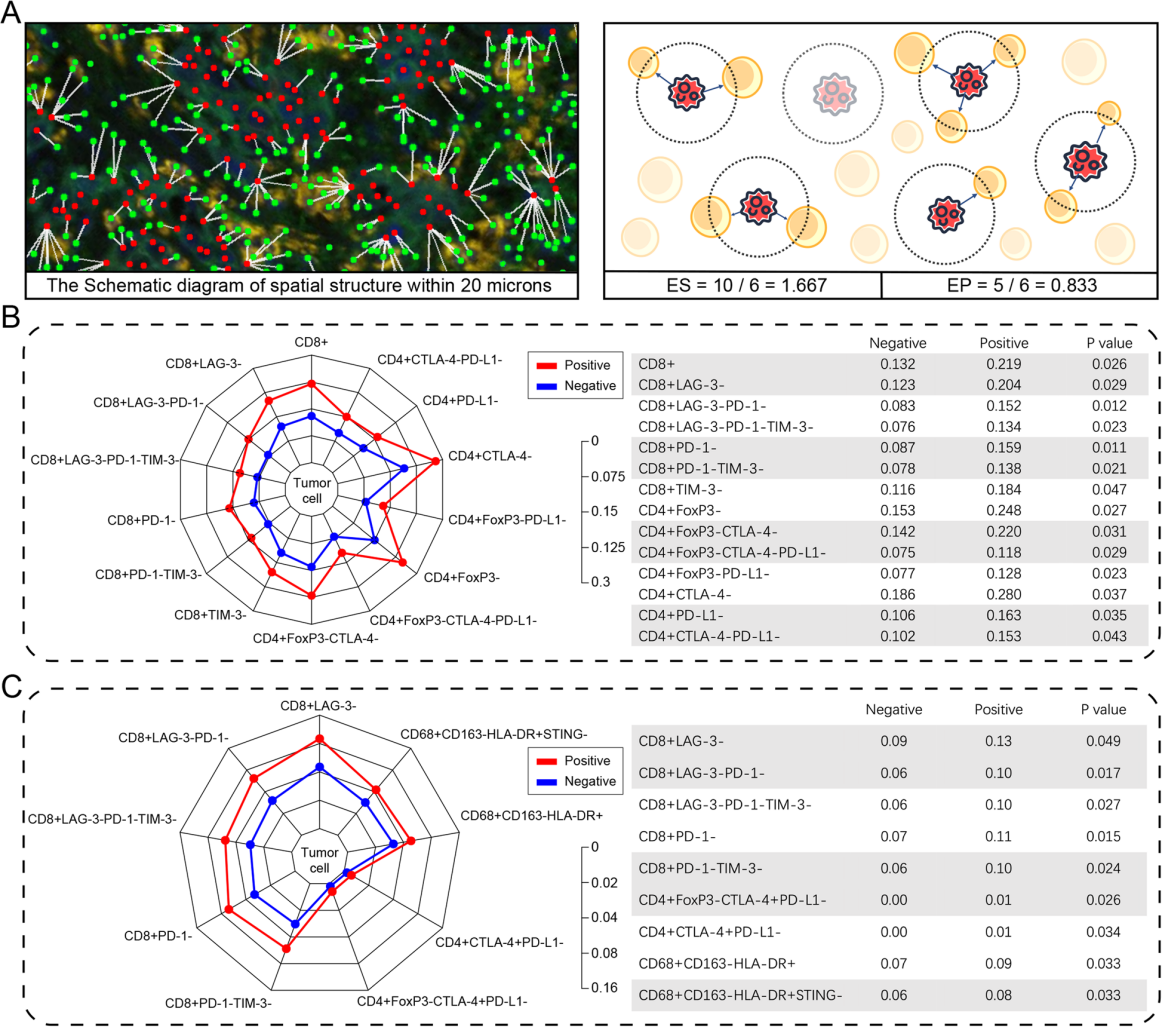
Unique spatial distribution of immune cell subtypes in CLDN18.2-positive GC. **A** Illustration of the spatial analysis involving tumor cell and immune cell. Red dots and green dots represent tumor cells and immune cells, respectively. White line connecting a red dot and a green dot means that the distance between two cells are less than 20 microns. ES: effective score. EP: effective percent. Comparison of the effective scores (**B**) and effective percent (**C**) between the CLDN18.2-positive group and CLDN18.2-negative group (central cells: tumor cells; peripheral cells: immune cells; radius range: 20 microns). Red points and lines: CLDN18.2-positive; blue points and lines: CLDN18.2-negative

Given our ability to precisely define the positions of individual tumor cells and immune cells, we sought to evaluate the spatial organization of these cells in GC. To further study localization patterns, we used a bioinformatics tool (pdist; see Methods) to determine the nucleus-to-nucleus distances between the two cell types. To incorporate both cell proximity and their numbers, effective scores and effective percentages were introduced (Fig. 4A). The radius (20 μm) was preselected in order to identify immune cell populations that were likely capable of effective cell-to-cell interaction with tumor cells, consistent with prior studies in GC.

As shown in Fig. 4B, in CLDN18.2-positive tumors, the effective scores of CD8^+^ T cells, CD8^+^LAG-3^−^ T cells, CD8^+^PD-1^−^ T cells, CD8^+^TIM-3^−^ T cells, CD4^+^FoxP3^−^ T cells, CD4^+^FoxP3^−^CTLA-4^−^ T cells, and CD4^+^FoxP3^−^PD-L1^−^ T cells were higher than those in CLDN18.2-negative tumors (0.22 vs. 0.13, *P* = 0.026; 0.20 vs. 0.12, *P* = 0.029; 0.16 vs. 0.087, *P* = 0.011; 0.18 vs. 0.12, *P* = 0.047; 0.25 vs. 0.15, *P* = 0.027; 0.22 vs. 0.14, *P* = 0.031; 0.13 vs. 0.077, *P* = 0.023, respectively). Comparison results of other immune cells were recorded in Additional file 1: Table S5. Higher effective percentages of CD8^+^LAG-3^−^ T cells, CD8^+^PD-1^−^ T cells, and CD68^+^CD163^−^HLA-DR^+^ (M1) macrophages were associated with higher CLDN18.2 expression (0.13 vs. 0.087, *P* = 0.049; 0.11 vs. 0.066, *P* = 0.015; 0.091 vs. 0.066, *P* = 0.033, respectively; Fig. 4C, Additional file 1: Table S6).

### Distinct microenvironment is associated with CLDN18.2-positivity in tumor-adjacent normal tissue

CLDN18.2-positive normal tissues showed a distinct immune microenvironment. CD4^+^FoxP3^+^ Treg immune cells, CD4^+^PD-L1^+^ T cells, and neutrophils were more abundantly observed in CLDN18.2-positive normal tissues (Fig. 3C, Additional file 2: Fig. S5A, Additional file 1: Table S7). In tumor-adjacent normal tissues, we also calculated the spatial organization between immune cells and epithelial cells. Unfortunately, we only found higher effective scores for CD8^+^PD-1^+^ T cells and neutrophils in CLDN18.2-positive normal tissue (Additional file 2: Fig. S5B, Additional file 1: Table S8-9). Furthermore, normal tissues with CLDN18.2-positivity showed a higher proportion of PD-1/PD-L1 expression on lymphocytes, suggesting distinct environmental features.

### The implications of tumor microenvironment in anti-PD-1/PD-L1 immunotherapy or CAR-T therapy

**Fig. 5:**
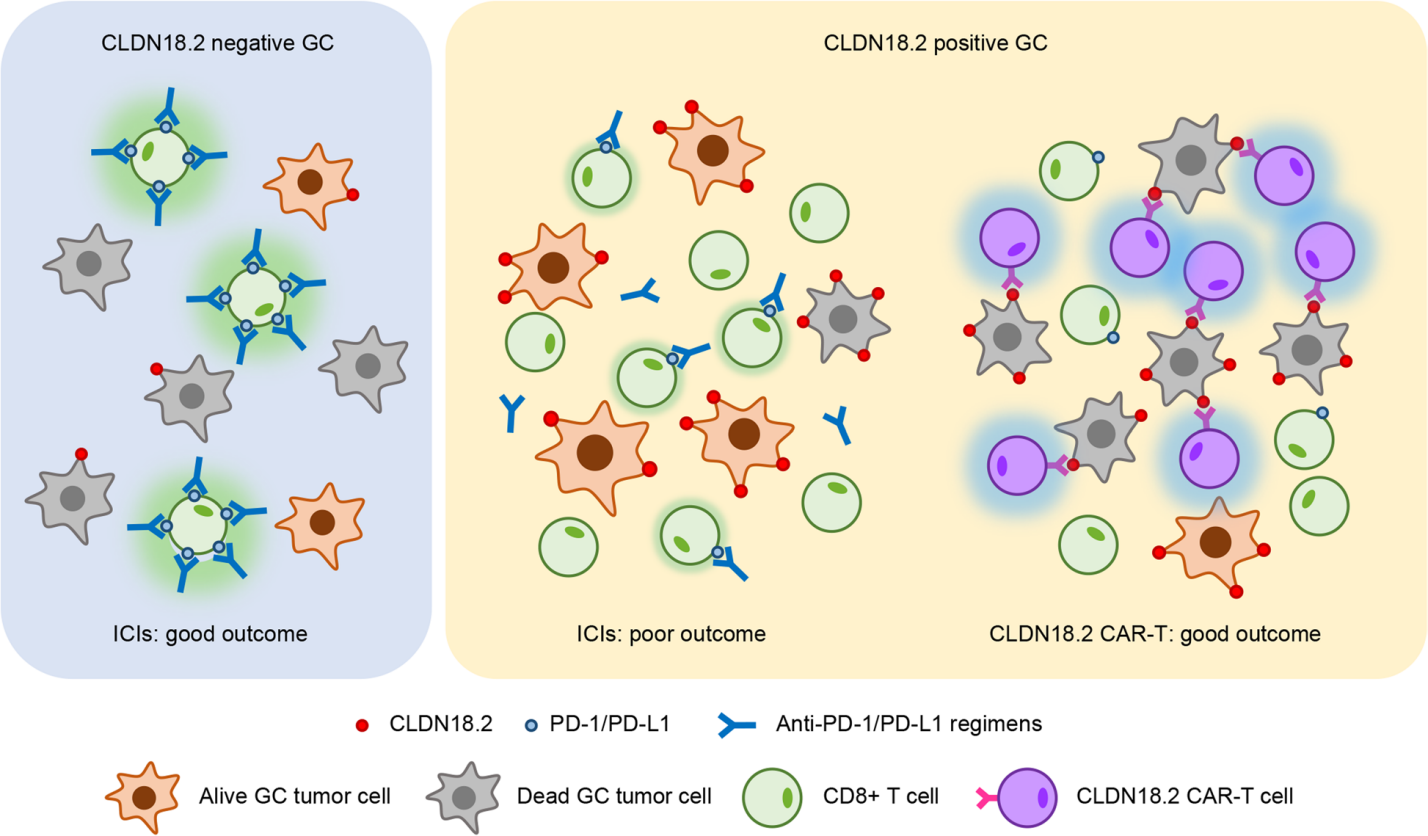
A graphical abstract for the major conclusions of the study. The immune microenvironmental features of GC stratified by CLDN18.2 indicated different therapeutic expectations to anti-PD-1/PD-L1 or CAR-T therapy. The blue area on the left represents the CLDN18.2-negative tumor microenvironment, where tumor cells expose few CLDN18.2 binding sites. The yellow area on the right represents the CLDN18.2-positive tumor microenvironment, where tumor cells expose more CLDN18.2 sites and CD8 + PD-1- T cells are enriched compared with CLDN18.2-negative gastric cancer

As shown in Fig. 5, in CLDN18.2-positive patients, the relatively lower expression of PD-1 in CD8^+^ T cells limits patients to benefit from PD-1/PD-L1 ICIs. CLDN18.2-targeted CAR-T cell therapy may be a promising treatment strategy in CLDN18.2-postive patients [12]. Tumor cells expose sufficient CLDN18.2 binding sites in CLDN18.2-positve patients and loose connection between tumor cells. These patients also harbor higher effective score and effective percent of non-exhausted CD8^+^ T cells and T helper indicating abundance active T cells to infiltrate in tumor core and exert cytotoxic functions.

## Discussion

CLDN18.2 is present in the tight junction supramolecular complex formed between normal epithelial cells and is retained during malignant transformation, making it an ideal candidate for antibody binding and immunity recruitment [6]. CLDN18.2 has the potential to become an important treatment molecule in patients with advanced GC and a trend of benefit in those with higher CLDN18.2 expression [8, 11]. Powered by multi-dimensional analysis, the main aim of this study was to investigate the immunotherapeutic outcomes and tumor microenvironmental features related to CLDN18.2 expression.

In this study, we reported that CLDN18.2 expression was correlated with advanced stage (III, IV). On the one hand, CLDN18.2 positivity is more common in advanced gastric cancer than in early (stage I and II) gastric cancer. On the other hand, based on the quantitative data from pathomics, we found that the average value of the proportion of CLDN18.2-positive tumor cells in advanced patients was also higher. The association between CLDN18.2 and advanced stage may be one of the reasons for the poor prognosis of CLDN18.2-positive gastric cancer, and poor/moderate-poor differentiation and diffused/mixed type should be considered as other reasons. In an Asian gastric cancer cohort of 3071 patients, almost all gastric cancers of diffuse/mixed type were poorly or moderately differentiated (1639/1648). Patients of diffuse/mixed type had a shorter median survival time of OS and disease-free survival (DFS) [27]. In our cohort, we found the GC of poor/mediate-poor differentiation had a higher proportion of CLDN18.2-positive tumor cells. Although the *P*-value is not statistically significant, a similar trend was also found in diffuse and mixed types. Above results explain the poor prognosis of CLDN18.2-positive gastric cancer from the perspective of differentiation and Lauren classification. A previous study showed that positive membrane CLDN18 expression was significantly associated with non-antral GCs (*P* = 0.016), diffuse type (Lauren classification) (*P* = 0.009), and EBV-associated cancers (*P* < 0.001) [28]. However, in a large Caucasian AGE/S cohort including 414 patients, high expression (immunoreactivity score > 8, detected in 17.2% patients) of CLDN18.2 was neither associated with clinicopathological features nor with OS [18]. CLDN18.2 was also highly expressed in gastric signet-ring cell carcinoma patients (SRCC) but was not a prognostic risk factor in advanced SRCC. Next generation sequencing found that GRIN2A mutation was related to CLDN18.2 expression in advanced gastric SRCC, which was frequently found in melanoma and induced the loss of tumor suppressor function [16]. These conflicting results might be due to different detection antibodies and various cut-off values.

In our study, EBV-positive GCs tended to show strong CLDN18.2 expression, which was consistent with the results in TCGA. Shinozaki et al. previously indicated a CLDN18-positive predominance in EBV-associated GCs [29]. Since preserved expression of CLDN18 was detected in immature gastric epithelium, our data support the hypothesis that EBV-associated GCs could arise directly from immature proliferating cells [28]. In our dataset, we found that the expression of CLDN18.2 (≥ 40%) was associated with inferior OS. Xu et al. reported that higher CLDN18 expression was correlated with perineural invasion and poor OS, which was consistent with our results [16].

Although the combination of immunotherapy with anti-CLDN18.2 has not been previously reported, a study pointed out that targeting CLDN18.2 could theoretically promote T cell infiltration and antigen presentation, which may influence the efficacy of immune checkpoint inhibitors [30]. Bispecific T cell engagers (BiTEs) against CD3 and CLDN18.2 can also redirect T cells towards the tumor target CLDN18.2 and induce T cell-mediated cell killing [31]. In this work, for the first time, we reported a negative association between CLDN18.2 expression and the prognosis of anti-PD-1/PD-L1 therapy. This correlation might be due to the unique tumor microenvironment of CLDN18.2-positive GC.

Whereas CD8^+^ subsets inform our understanding of the mechanism of action of these agents and associated T cell biology, a distilled panel of markers providing comparable prognostic accuracy is desirable for clinical assays. In the tumor core, we found that the CLDN18.2-positive group showed significantly higher total number of CD8^+^ T, CD8^+^LAG-3^−^ T, CD8^+^PD-1^−^ T, and CD8^+^TIM-3^−^ T cells than in the CLDN18.2-negative group. LAG-3 and TIM-3 are novel immune checkpoints that are co-expressed and co-regulated with other immune checkpoints such as PD-1 on CD8+ T cells [32, 33]. Preclinical studies have shown that targeting the TIM-3 and PD-1 pathways can reverse T cell exhaustion and regain antitumor immunity. Dosage and safety of the combined therapy are being explored in relevant phase I clinical trials [34–36]. A phase 2–3, global, double-blind, randomized clinical trial evaluated the PFS of relatlimab (a LAG-3–blocking antibody) and nivolumab as a fixed-dose combination compared with nivolumab alone in melanoma. The median PFS was 10.1 months with relatlimab–nivolumab and 4.6 months (95% CI, 3.4 to 5.6) with nivolumab [37]. Yujun Park et al. clustered 58 gastric cancers based on PD-1, LAG-3, and TIM-3. They found that the subgroups with low expression of immune checkpoints (cluster 1) had poor prognosis, which is consistent with the results for CLDN18.2-positive GC in our cohort [38]. However, the mechanism of insufficient immune responses of PD-1, LAG-3 and TIM-3 in CLDN18.2-positive GC still needs further investigation. Additionally, a total number of CD4^+^ T, CD4^+^FoxP3^−^ T, CD4^+^FoxP3^−^CTLA-4^−^ T, and CD4^+^FoxP3^−^PD-L1^−^ T cells was significantly higher in the CLDN18.2-positve group than that in the CLDN18.2-negative group. To our knowledge, this is the first study to reveal the association of higher level of CD8^+^ and CD4^+^ T cells with CLDN18.2-positive GC tumors. Furthermore, in spatial organization analysis, we also observed higher effective scores for CD8^+^ T, CD8^+^LAG-3^−^ T, CD8^+^PD-1^−^ T, CD8^+^TIM-3^−^ T, CD4^+^FoxP3^−^ T, CD4^+^FoxP3^−^CTLA-4^−^ T, and CD4^+^FoxP3^−^PD-L1^−^ T cells in CLDN18.2-positive tumors compared with CLDN18.2-negative tumors, suggesting a higher possibility of tumor-immune cell interaction.

The spatial distribution of cells in the tumor microenvironment is considered to be one of the major reasons for the spatial heterogeneity of tumors and is associated with prognosis in pancreatic cancer and gastric cancer [19, 39]. The spatial proximity of tumor cells and immune cells represents an increased likelihood of cell-cell contact. Compared with only counting the different cell types, spatial analysis focus on both the relative numbers of central and peripheral cells, as well as their relative positions [22]. The effective score indicates that there are more CD8^+^PD-1^−^ T cells, CD8^+^LAG-3^−^ T cells, and CD8^+^TIM-3^−^ T cells around tumor cells, so the immunosuppressive effect based on immune checkpoints may not be the main reason of immune escape in CLDN18.2-positive gastric cancer. CLDN18.2-targeted CAR-T cell therapy guides immune effector T cells to recognize tumor cells through the binding of ligands and receptors [40]. The higher effective percent in this study suggested that more tumor cells were surrounded by non-exhausted CD8^+^ T cells, which will benefit CAR-T cell therapy. The above results support the potential of CAR-T therapy in CLDN18.2-positive gastric cancer.

Collectively, higher rate and effective score of CD8+, CD4+ total, and effector T cells represented a unique tumor immune environment. CLDN18.2-positive GC contained relatively less PD-1/PD-L1 expressing CD8/CD4 T cells leads to the inferior survival in patients receiving anti-PD-1/anti-PD-L1 treatment. In contrast, the promising results of anti-CLDN18.2 therapy with anti-CD3 BiTEs or CAR-T therapy could be due to assist T cell-mediated cell killing. It is worth mentioning that in our clinical practice, adverse effects (such as cytokine release syndrome or discomfort in the digestive tract) were observed more frequently for CLDN18.2-antibodies and moderately for CAR-T therapy. The difference in microenvironment between tumor and tumor-adjacent normal tissue may explain this difference in adverse events. In tumor-adjacent normal tissue, CLDN18.2-postive showed a distinct tumor microenvironment with higher CD4^+^ FoxP3^+^ Treg immune cells and CD4^+^PD-L1^+^ T cells. The immune response in tumor-adjacent normal tissues is not as intense as that in tumor tissues. The relatively suppressive immune microenvironment in normal patients may be the cause of tumor recurrence. Our previous study indicated that NOD/SCID mice treated with CLDN18.2-targeted CAR-T cells displayed no obvious toxicity in normal stomach tissues, which might be ascribed to the limited exposure of CAR binding epitopes in normal tissues or the normal tissue distinct microenvironment [11].

The limitation of this study is that all tumor samples were obtained from a single institution. However, as a tier one cancer hospital in China, our patients were collected from all over the country, which increases the possibility of representing the overall patient population. Moreover, our results show CLDN18.2 expression in Asian patients with GC is very similar to that in the patients included in the FAST trial. Therefore, these data provide strong support for CLDN18.2 as a promising target. CLDN18.2-positive GC displayed unique clinicopathological and immune-microenvironmental characteristics, which provided a phenotypic view of the biological role of CLDN18.2 GC. With well-depicted molecular profiles and well-designed clinical trials to be presented soon, we believe that anti-CLDN18.2 therapies will bring promising results for GC in the future.

## Conclusions

In conclusion, CLDN18.2-positive GC harbors unique immune-microenvironmental characteristics, which endows CLDN18.2 targeted CAR-T as a promising treatment strategy and warrants further investigation in clinical practice.

### Supplementary Information

**Additional file 1: Table S1.** Details of antibodies used for multiple immunohistochemistry. **Table S2.** The clinical, pathological, and molecular characteristics of groups with higher or lower CLDN18 expression in TCGA cohorts. **Table S3.** The clinical, pathological, and molecular characteristics of CLDN18.2 positive or negative gastric cancer patients receiving immunotherapy. **Table S4.** The expression proportion of cell markers in CLDN18.2 positive and negative tumor tissues. **Table S5.** The effective score of cell markers in CLDN18.2 positive and negative tumor tissues. **Table S6.** The effective percent of cell markers in CLDN18.2 positive and negative tumor tissues. **Table S7.** The expression proportion of cell markers in CLDN18.2 positive and negative normal tissues. **Table S8.** The effective score of cell markers in CLDN18.2 positive and negative normal tissues. **Table S9.** The effective percent of cell markers in CLDN18.2 positive and negative normal tissues.

**Additional file 2: Figure S1.** CLDN18.2 expression in GC. Representative images of IHC-stained GC tissues with different CLDN18.2 intensity. **Figure S2.** The relationship between the proportion of CLDN18.2 expression and prognosis. (A) AUC curve of predicting the efficacy of anti-PD-1/PD-L1 regimens in GC based on the proportion of moderate-to-strong CLDN18.2 expression; (B) OS, irOS and irPFS of GC patients were stratified by Log-Rank test based on the proportion of moderate-to-strong CLDN18.2 expression in normal. (C) OS, irOS and irPFS of GC patients were stratified by Log-Rank test based on the balance of the proportion of moderate-to-strong CLDN18.2 expression in GC and matched normal tissues. **Figure S3.** The correlation of CLDN18.2 with other immune checkpoint markers in GC. "Corr" in the grey text represents the correlation coefficients in all patients. "Negative" in the green text represents the correlation coefficient in the CLDN18.2 negative group. "Positive" in the red text represents the correlation coefficient in the CLDN18.2 positive group. In histograms, dot plots, and density plots, red represents CLDN18.2-positive and green represents CLDN18.2-negativity. * *P* < 0.05, ** *P* < 0.01, and *** *P* < 0.001. **Figure S4.** The detailed immune composition according to CLDN18.2 in GC. (A) The abundance of CD8+ T cells according to CLDN18 classification in TCGA. Student t-test. * *P* < 0.05, ** *P* < 0.01, *** *P* < 0.001 and not significant (ns). The comparison of M1 and M2 macrophages in our cohort (B) and TCGA (C) based on CLDN18.2 classification. * *P* < 0.05, ** *P* < 0.01, *** *P* < 0.001 and not significant (ns). (D) An appendix heatmap presenting the rate of immune cell subtypes in TC. **Figure S5.** Quantitative and spatial information on immune cells in GC or adjacent normal tissues. (A) An appendix heatmap presenting the rate of immune cell subtypes in adjacent normal tissues. Effective score (B) and effective percent (C) in adjacent normal samples grouped by CLDN18.2 expression (central cells: tumor cells; peripheral cells: immune cells; radius range: 20 microns).

## Data Availability

The datasets used and/or analyzed during the current study are available from the corresponding author on reasonable request.

## Abbreviations

GC: Gastric cancer
m-IHC: Multiplex immunohistochemistry
CLDN18.2: Isoform 2 of claudin-18
ADCC: Antibody-dependent cellular cytotoxicity
CDC: Complement-dependent cytotoxicity
ORR: Objective response rate
PFS: Progression-free survival
OS: Overall survival
HR: Hazard ratio
CAR: Chimeric antigen receptor
FFPE: Formalin-fixed, paraffin-embedded
MMR: Mismatch repair
ISH: In situ hybridization
EBER1: Epstein-Barr-encoded RNA 1
CR: Complete response
PR: Partial response
PD: Progressive disease
SD: Stable disease
TSA: Tyramine signal amplification
TC: Tumor core
N: Normal
IM: Invasion margin
ROI: Region of interest
TCGA: The Cancer Genome Atlas
PSI: Percent-splice-in
ANOVA: Analysis of variance
TIICs: Tumor-infiltrating immune cells
CPS: Combined positive score
SRCC: Signet-ring cell carcinoma
