# Correction: Multiplex immunohistochemistry defines the tumor immune microenvironment and immunotherapeutic outcome in CLDN18.2-positive gastric cancer

**DOI:** 10.1186/s12916-023-03090-4

**Published:** 2023-09-29

**Authors:** Keren Jia, Yang Chen, Yu Sun, Yajie Hu, Lei Jiao, Jie Ma, Jiajia Yuan, Changsong Qi, Yanyan Li, Jifang Gong, Jing Gao, Xiaotian Zhang, Jian Li, Cheng Zhang, Lin Shen

**Affiliations:** 1 Department of Gastrointestinal Oncology, Key Laboratory of Carcinogenesis and Translational Research (Ministry of Education), Peking University Cancer Hospital & Institute, Beijing, China; 2 Department of Pathology, Key Laboratory of Carcinogenesis and Translational Research (Ministry of Education), Peking University Cancer Hospital & Institute, Beijing, China; 3 Panovue Biotechnology (Beijing) Co, Ltd, Beijing, China; 4 National Cancer Center/National Clinical Research Center for Cancer/Cancer Hospital & Shenzhen Hospital, Chinese Academy of Medical Sciences and Peking Union Medical College, Beijing, China

**Correction: BMC Medicine 20, 223 (2022)**

**https://doi.org/10.1186/s12916-022-02421-1**

The original article [1] contains numerical errors in the following sentence regarding median irOS:

‘Additionally, CLDN18.2-positive group had shorter OS and irOS than CLDN18.2-negative group (median OS: 23.33 vs.36.6 months, P < 0.001; median irOS: 10.03 vs. 20.13 months, P = 0.044, respectively).’

The sentence should instead read as follows:

‘Additionally, CLDN18.2-positive group had shorter OS and irOS than CLDN18.2-negative group (median OS: 23.33 vs.36.6 months, P < 0.001; median irOS: 9.27 vs. 12.83 months, P = 0.044, respectively).’'
