# Supplementary material for: Multiplex immunohistochemistry defines the tumor immune microenvironment and immunotherapeutic outcome in CLDN18.2-positive gastric cancer
Source: BMC Med. 2022 Jul 11;20:223. doi: 10.1186/s12916-022-02421-1 (PMC9272556; doi:10.1186/s12916-022-02421-1)
Supplement: Supplementary file 2 — Additional file 2: Figure S1. CLDN18.2 expression in GC. Representative images of IHC-stained GC tissues with different CLDN18.2 intensity. Figure S2. The relationship between the proportion of CLDN18.2 expression and prognosis. (A) AUC curve of predicting the efficacy of anti-PD-1/PD-L1 regimens in GC based on the proportion of moderate-to-strong CLDN18.2 expression; (B) OS, irOS and irPFS of GC patients were stratified by Log-Rank test based on the proportion of moderate-to-strong CLDN18.2 expression in normal. (C) OS, irOS and irPFS of GC patients were stratified by Log-Rank test based on the balance of the proportion of moderate-to-strong CLDN18.2 expression in GC and matched normal tissues. Figure S3. The correlation of CLDN18.2 with other immune checkpoint markers in GC. "Corr" in the grey text represents the correlation coefficients in all patients. "Negative" in the green text represents the correlation coefficient in the CLDN18.2 negative group. "Positive" in the red text represents the correlation coefficient in the CLDN18.2 positive group. In histograms, dot plots, and density plots, red represents CLDN18.2-positive and green represents CLDN18.2-negativity. * P < 0.05, ** P < 0.01, and *** P < 0.001. Figure S4. The detailed immune composition according to CLDN18.2 in GC. (A) The abundance of CD8+ T cells according to CLDN18 classification in TCGA. Student t-test. * P < 0.05, ** P < 0.01, *** P < 0.001 and not significant (ns). The comparison of M1 and M2 macrophages in our cohort (B) and TCGA (C) based on CLDN18.2 classification. * P < 0.05, ** P < 0.01, *** P < 0.001 and not significant (ns). (D) An appendix heatmap presenting the rate of immune cell subtypes in TC. Figure S5. Quantitative and spatial information on immune cells in GC or adjacent normal tissues. (A) An appendix heatmap presenting the rate of immune cell subtypes in adjacent normal tissues. Effective score (B) and effective percent (C) in adjacent normal samples grouped by CLDN18.2 expres [file 12916_2022_2421_MOESM2_ESM.pdf]

Additional file 2

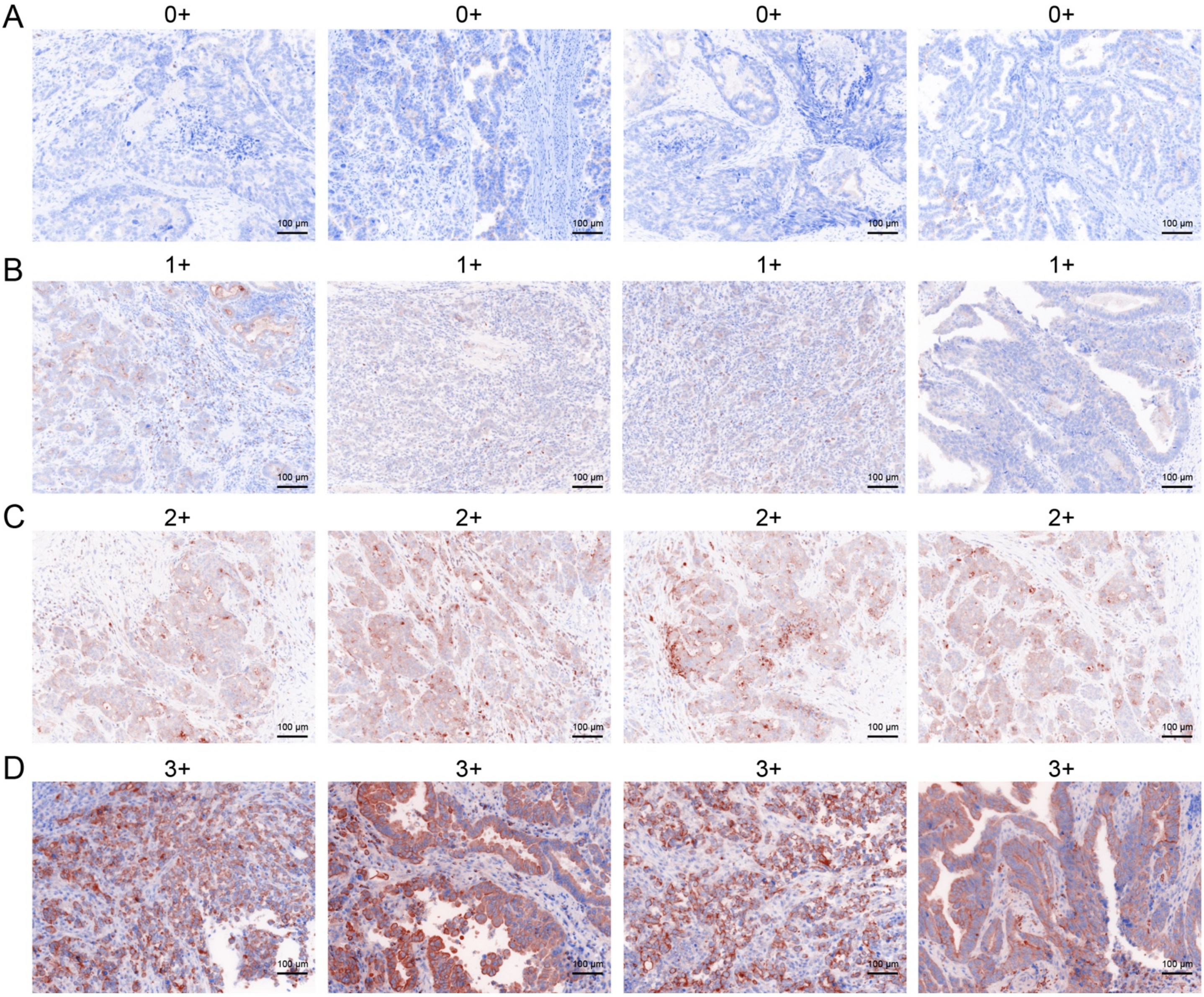

Additional file 2: Fig. S1. CLDN18.2 expression in GC

Representative images of IHC-stained GC tissues with different CLDN18.2 intensity.

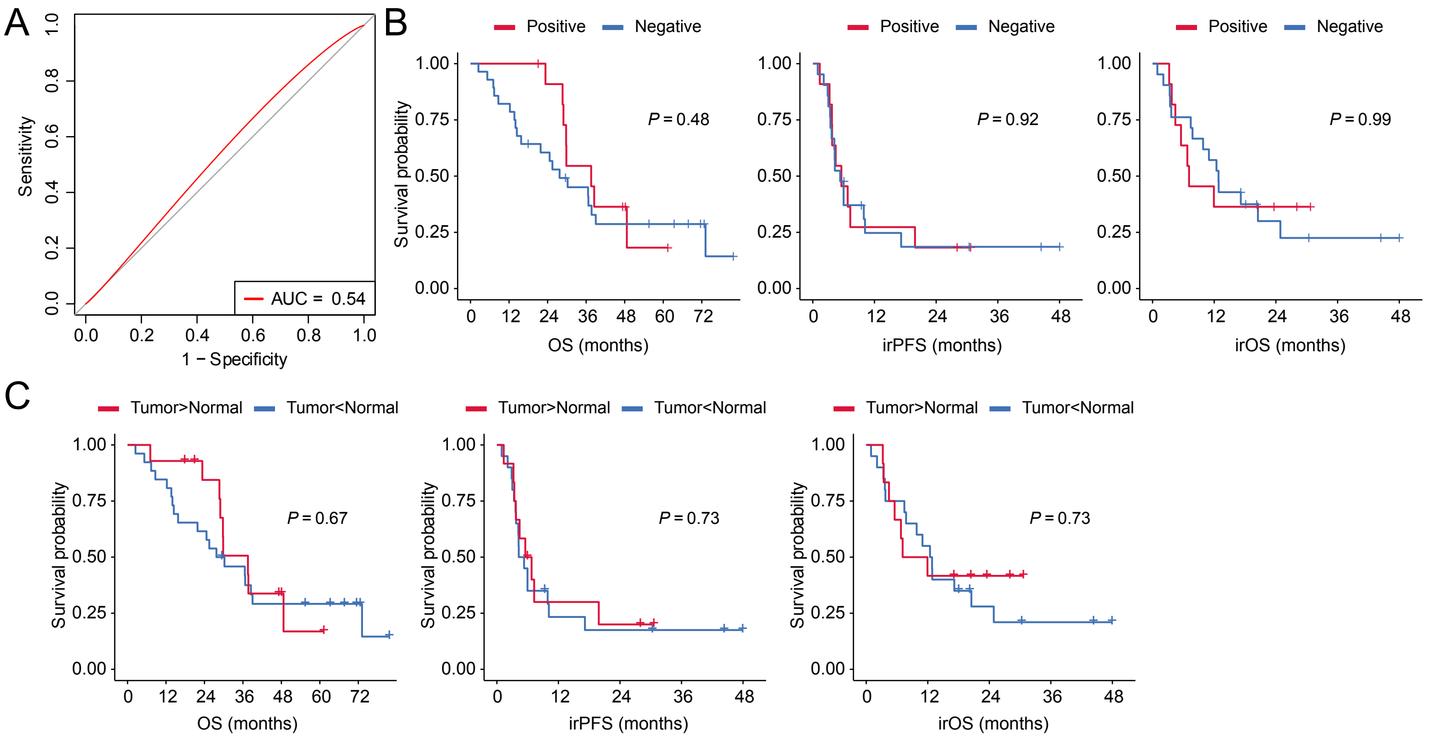

Additional file 2: Fig. S2. The relationship between the proportion of CLDN18.2 expression and prognosis

(A) AUC curve of predicting the efficacy of anti-PD-1/PD-L1 regimens in GC based on the proportion of moderate-to-strong CLDN18.2 expression;

(B) OS, irOS and irPFS of GC patients were stratified by Log-Rank test based on the proportion of moderate-to-strong CLDN18.2 expression in normal.

(C) OS, irOS and irPFS of GC patients were stratified by Log-Rank test based on the balance of the proportion of moderate-to-strong CLDN18.2 expression in GC and matched normal tissues.

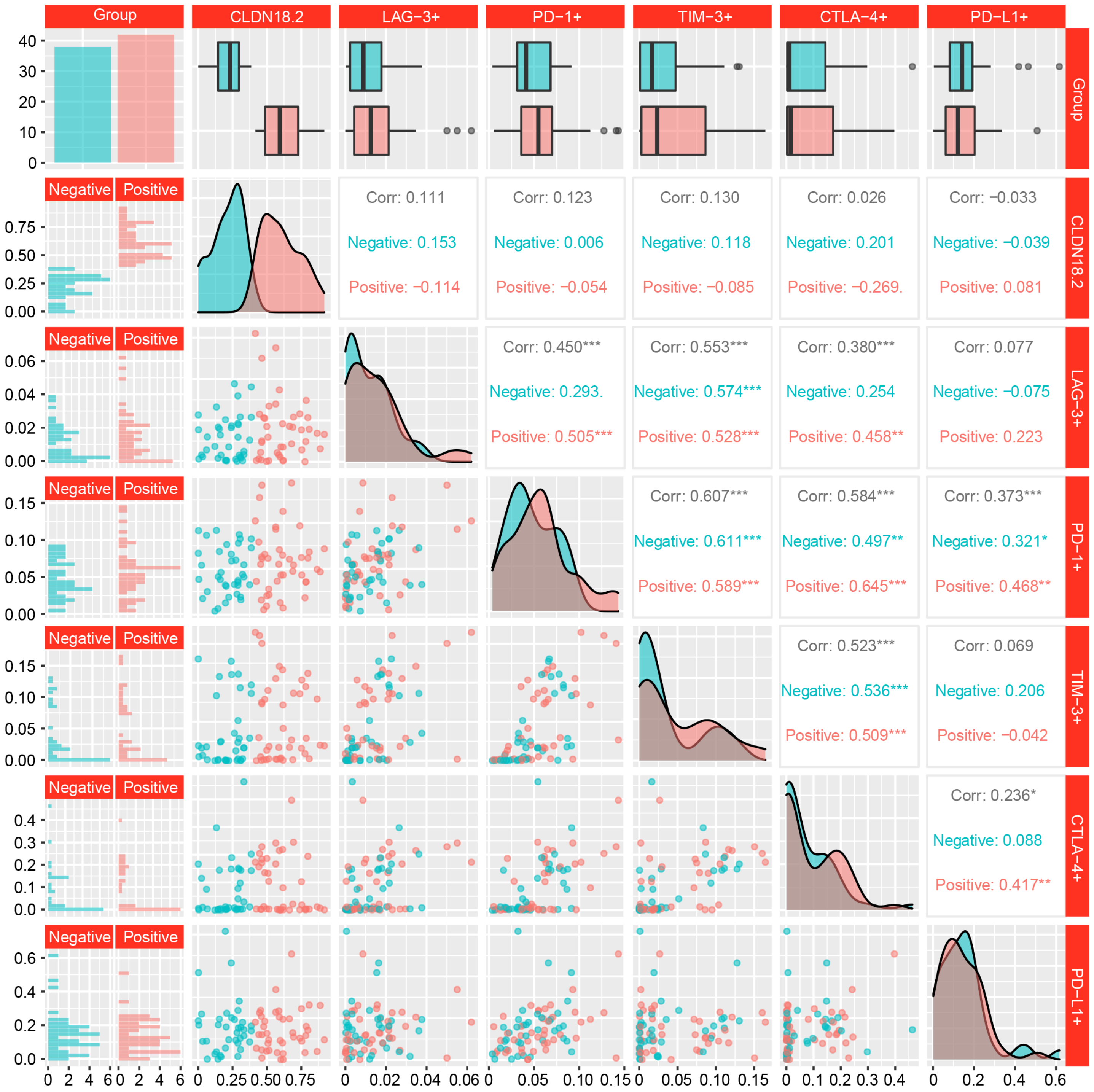

Additional file 2: Fig. S3. The correlation of CLDN18.2 with other immune checkpoint markers in GC

"Corr" in the grey text represents the correlation coefficients in all patients. "Negative" in the green text represents the correlation coefficient in the CLDN18.2 negative group. "Positive" in the red text represents the correlation coefficient in the CLDN18.2 positive group. In histograms, dot plots, and density plots, red represents CLDN18.2-positive and green represents CLDN18.2-negativity. * $P < 0.05$, ** $P < 0.01$, and *** $P < 0.001$.

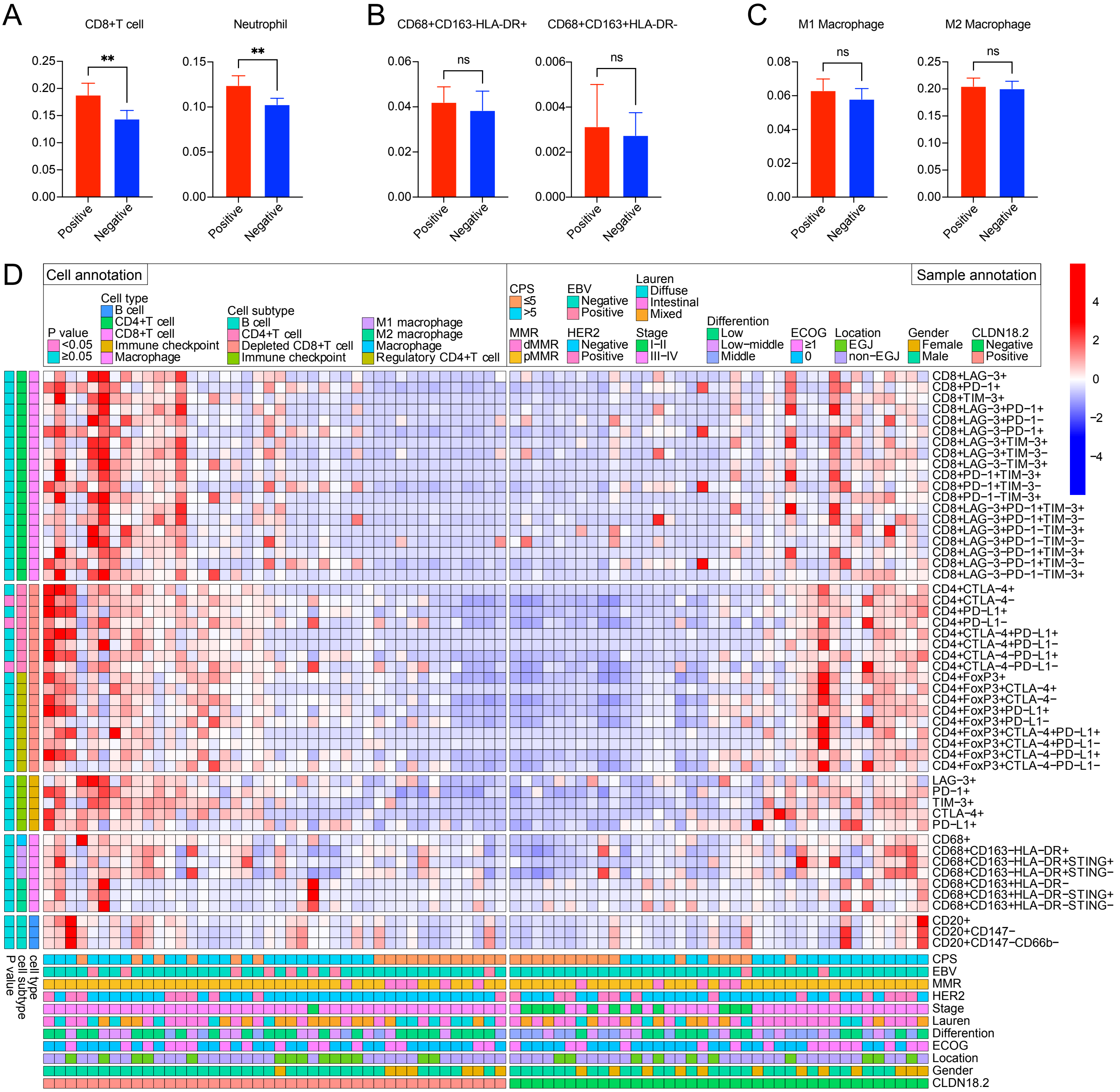

Additional file 2: Fig. S4. The detailed immune composition according to CLDN18.2 in GC

(A) The abundance of CD8+ T cells according to CLDN18 classification in TCGA. Student t-test. * $P < 0.05$, ** $P < 0.01$, *** $P < 0.001$ and not significant (ns).

The comparison of M1 and M2 macrophages in our cohort (B) and TCGA (C) based on CLDN18.2 classification. * $P < 0.05$, ** $P < 0.01$, *** $P < 0.001$ and not significant (ns).

(D) An appendix heatmap presenting the proportion of immune cell subtypes in TC.

A

B

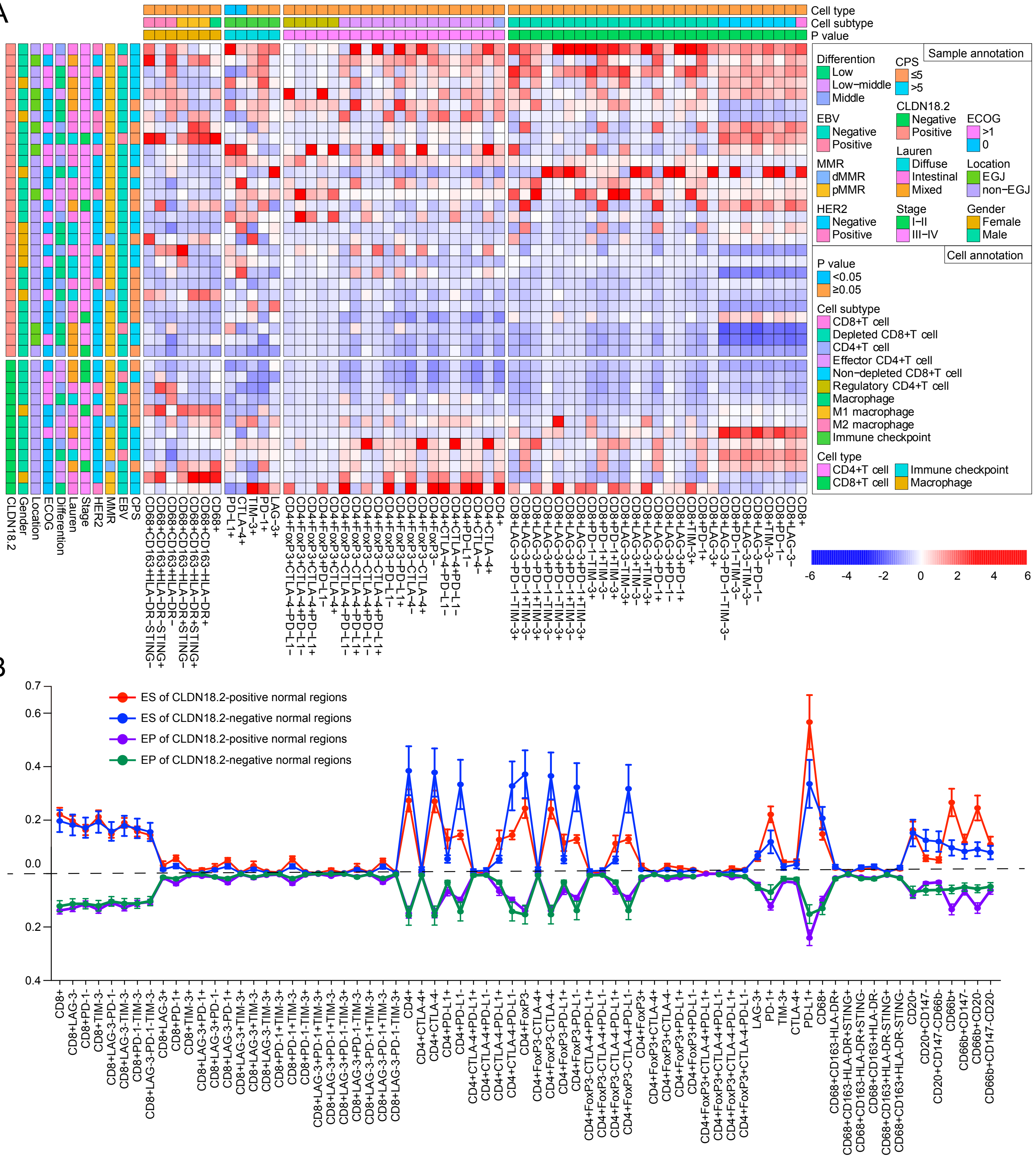

Additional file 2: Fig. S5. Quantitative and spatial information on immune cells in GC or adjacent normal tissues.

(A) An appendix heatmap presenting the proportion of immune cell subtypes in adjacent normal tissues. Effective score (B) and effective percent (C) in adjacent normal samples grouped by CLDN18.2 expression (central cells: tumor cells; peripheral cells: immune cells; radius range: 20 microns).
